# Standardized Cannabis Smoke Extract Induces Inflammation in Human Lung Fibroblasts

**DOI:** 10.3389/fphar.2022.852029

**Published:** 2022-03-28

**Authors:** Noof Aloufi, Yoon Namkung, Hussein Traboulsi, Emily T. Wilson, Stephane A. Laporte, Barbara L.F. Kaplan, Matthew K. Ross, Parameswaran Nair, David H. Eidelman, Carolyn J. Baglole

**Affiliations:** ^1^ Meakins-Christie Laboratories, Montreal, QC, Canada; ^2^ Translational Research in Respiratory Diseases Program at the Research Institute of the McGill University Health Centre, Montreal, QC, Canada; ^3^ Department of Pathology, McGill University, Montreal, QC, Canada; ^4^ Department of Medical Laboratory Technology, Applied Medical Science, Taibah University, Medina, Saudi Arabia; ^5^ Department of Medicine, McGill University, Montreal, QC, Canada; ^6^ Department of Pharmacology and Therapeutics, McGill University, Montreal, QC, Canada; ^7^ Department of Comparative Biomedical Sciences, Mississippi State University, Mississippi State, MS, United States; ^8^ Department of Medicine, McMaster University and St Joseph’s Healthcare, Hamilton, ON, Canada

**Keywords:** fibroblast, inflammation, cannabis smoke, thc, cbd, lungs, CB1, BRET

## Abstract

Cannabis (marijuana) is the most commonly used illicit product in the world and is the second most smoked plant after tobacco. There has been a rapid increase in the number of countries legalizing cannabis for both recreational and medicinal purposes. Smoking cannabis in the form of a joint is the most common mode of cannabis consumption. Combustion of cannabis smoke generates many of the same chemicals as tobacco smoke. Although the impact of tobacco smoke on respiratory health is well-known, the consequence of cannabis smoke on the respiratory system and, in particular, the inflammatory response is unclear. Besides the combustion products present in cannabis smoke, cannabis also contains cannabinoids including Δ^9^-tetrahydrocannabinol (Δ^9^-THC) and cannabidiol (CBD). These compounds are hydrophobic and not present in aqueous solutions. In order to understand the impact of cannabis smoke on pathological mechanisms associated with adverse respiratory outcomes, the development of *in vitro* surrogates of cannabis smoke exposure is needed. Therefore, we developed a standardized protocol for the generation of cannabis smoke extract (CaSE) to investigate its effect on cellular mechanisms *in vitro*. First, we determined the concentration of Δ^9^-THC, one of the major cannabinoids, by ELISA and found that addition of methanol to the cell culture media during generation of the aqueous smoke extract significantly increased the amount of Δ^9^-THC. We also observed by LC-MS/MS that CaSE preparation with methanol contains CBD. Using a functional assay in cells for CB1 receptors, the major target of cannabinoids, we found that this CaSE contains Δ^9^-THC which activates CB1 receptors. Finally, this standardized preparation of CaSE induces an inflammatory response in human lung fibroblasts. This study provides an optimized protocol for aqueous CaSE preparation containing biologically active cannabinoids that can be used for *in vitro* experimentation of cannabis smoke and its potential impact on various indices of pulmonary health.

## Introduction

Cannabis has been used for medical purposes for thousands of years (Hillig, 2005; Rana, 2010; Atakan, 2012). Cannabis, commonly referred as marijuana, is a flowering plant belonging to the family *Cannabaceae*. There are three main subspecies of cannabis: *C. sativa*, *C. indica* and *C. ruderalis*, which are differentiated by key physical characteristics and production of cannabinoids (Hillig, 2005; Rana, 2010; Atakan, 2012). Cannabis produces more than 100 cannabinoids (Baron, 2018) that have many effects in the human body, including modulation of mood, memory and the immune response. One of the major cannabinoids is Δ^9^-tetrahydrocannabinol (Δ^9^-THC), which is responsible for the psychotropic effect of cannabis via activation of cannabinoid-1 (CB1) receptors in the brain (Mersiades et al., 2018). Cannabidiol (CBD), cannabigerol (CBG) and cannabichromene (CBC) are other cannabinoids currently under scientific investigation for their therapeutic potential. Of these, CBD has gained the most interest, particularly as an anti-inflammatory agent that lacks the psychoactive properties of Δ^9^-THC (Rajan et al., 2016; Morales et al., 2017).

Δ^9^-THC and CBD are produced in the trichomes of the female inflorescence as acidic precursors THCA and CBDA, respectively, that undergo decarboxylation when heated by consumption methods such as smoking (Tahir et al., 2021). According to the World Health Organization (WHO), approximately 15 million people (3% of world population) consume cannabis each year, making this the most widely-used illicit drug in the world. Currently, cannabis is the second most-smoked plant after tobacco (Baron, 2018; Brown, 2020; Campeny et al., 2020; Li et al., 2020), making inhalation of cannabis smoke the most common consumption method (Schuermeyer et al., 2014). Smoking cannabis provides the fastest Δ^9^-THC delivery to the body, resulting in rapid onset of psychoactive effects. Like tobacco smoke, cannabis smoke also contains carcinogens [e.g., polycyclic aromatic hydrocarbons (PAHs)] and other toxicants (e.g., carbon monoxide) (Moir et al., 2008; Maertens et al., 2009; Graves et al., 2020). A recent study showed that there are 4,350 and 2,575 compounds in tobacco and cannabis smoke, respectively. Of these, 69 were common in both and are known to have adverse health risks through carcinogenic, mutagenic, or other toxic mechanisms (Graves et al., 2020). Unlike tobacco smoke, where the adverse respiratory effects are well-established (Strzelak et al., 2018), there are significant gaps in our understanding of the impact of cannabis smoke on respiratory health. Based on a limited number of studies, there is evidence that cannabis smoking is associated with inflammation and chronic bronchitis (Yayan and Rasche, 2016; Urban and Hureaux, 2017). Cannabis smoke can also negatively affect physical (e.g., mucociliary clearance) and immunological respiratory defense mechanisms (Chatkin et al., 2017). Regular cannabis use may also increase risk for asthma and accelerate the decline in lung function (Chatkin et al., 2019). However, the net effects of cannabis smoke on respiratory health, and in particular inflammation, remain largely unknown and such findings are often complicated by concurrent tobacco use in human participants. Thus, there is a pressing need to understand the consequences of cannabis smoke on the inflammatory response.

Our understanding of the ill health effects of tobacco smoke were driven in part by preclinical models of exposure. There are now established *in vitro* and *in vivo* models that recapitulate many of the exposure parameters observed in humans. These models have been extensively used to evaluate the mechanistic impact of tobacco smoke exposure (Carp and Janoff, 1978; Aoshiba et al., 2001; Carnevali et al., 2003; Baglole et al., 2006; Damico et al., 2011; Zago et al., 2013; de Souza et al., 2014; Guerrina et al., 2021a; Rico de Souza et al., 2021). Of these, cigarette smoke extract (CSE) is a widely-utilized *in vitro* surrogate for tobacco smoke exposure, and protocols for the generation of CSE are established and readily adaptable by many laboratories (Carp and Janoff, 1978; Martey et al., 2005; Baglole et al., 2006; Baglole et al., 2008b; Bertram et al., 2009; Damico et al., 2011). However, no such standardized protocol for cannabis smoke extract (CaSE) currently exists, greatly limiting investigation into the impact of cannabis smoke on biological and toxicological indices. Therefore, we developed a standardized protocol for the preparation of an aqueous cannabis smoke extract (CaSE) for *in vitro* evaluation. We used a legal cannabis source with a described composition and developed a protocol for standardization that allows for comparison between studies; this CaSE can be prepared and standardized using common laboratory equipment. Importantly, we confirmed that these CaSE preparations contain pharmacologically active Δ^9^-THC using a signaling pathway downstream of the CB1 receptor: the Rho small G protein, with a Bioluminescence Resonance Energy Transfer (BRET) assay for this effector (Namkung et al., 2018). Finally, we used CaSE to show that key inflammatory markers are induced in human lung cells, suggesting that cannabis smoke is not harmless. With more countries legalizing cannabis for medical purposes, additional research is needed to better understand the cellular and molecular consequences of cannabis smoke exposure.

## Materials and Methods

### Chemicals

All chemicals were obtained from Sigma (St. Louis, MO) unless otherwise indicated. Coelenterazine 400a was purchased from Nanolight™ Technology. 2-AG, Δ^9^-THC and CBD are from Cayman Chemical (Ann Arbor, MI). The sp-hCB1 encoding plasmid (signal peptide human CB1) was a gift from Michel Bouvier, (University of Montreal).

### Preparation of Cigarette Smoke Extract (CSE)

Research grade cigarettes (3R4F) with a filter were acquired from the Kentucky Tobacco Research Council (Lexington, KY). Research grade cigarettes (3R4F) contain 0.73 mg of nicotine, 9.4 mg of tar, and 12.0 mg of CO as described by the manufacturer. CSE was produced as previously described by us (Baglole et al., 2008a; Zago et al., 2013; Guerrina et al., 2021a; Guerrina et al., 2021b). Briefly, CSE was prepared by bubbling smoke from a cigarette through 10 ml of serum-free cell culture medium with the exception that some extracts were prepared with 30% methanol (MeOH). The CSE was then sterile-filtered with a 0.45-μm filter (25-mm Acrodisc; Pall Corp., Ann Arbor, MI). Standardization was done for each CSE preparation by spectrophotometer using an OD_320_ nm of 0.65 to represent 100% CSE as described (Baglole et al., 2006; Zago et al., 2013).

### Preparation of Cannabis Smoke Extract

Cannabis was purchased from the *Société québécoise du cannabis* SQDC online store (Quebec, Canada). Whole flower cannabis that was selected for purchase contained varying cannabinoid profiles based on THC/CBD content. Those purchased were as follows: 1) Indica-THC dominant; contains 16–22% THC and 0–0.1% CBD (#688083002311). 2): Sativa-CBD dominant; contains 0.1–2% THC and 13–19% CBD (#694144000219) and 3) Hybrid-Balanced: contains 5–11% THC and 5–11% CBD (#688083002588). Cannabis joints (cigarettes) were hand-rolled by grinding the dried cannabis flower with a plastic grinder and packing the product into classic 1 1/4 size rolling paper (RAW^®^). Each cannabis cigarette contained 0.5 ± 0.05 g of cannabis. A slim unrefined cellulose filter (RAW^®^) was added to the end of the joint. Then, CaSE was produced as previously described for CSE (Baglole et al., 2008a; Zago et al., 2013; Guerrina et al., 2021b) where the smoke from the lit cannabis cigarette was bubbled through 10ml of serum-free cell culture Dulbecco’s modified Eagle’s medium (DMEM) with or without 30% methanol (MeOH) or 30% ethanol (EtOH). CaSE was then filtered using a 0.45-μm filter (25-mm Acrodisc; Pall Corp., Ann Arbor, MI). Because the tar components in tobacco and cannabis are similar (Tashkin, 2013), and chemical species of tobacco tar absorb light at 320 nm (Taylor et al., 2020), we standardized each CaSE preparation as previously described for CSE (Baglole et al., 2008a; Zago et al., 2013; Guerrina et al., 2021a; Guerrina et al., 2021b) to ensure consistency in CaSE preparations between experiments. Similar to CSE preparation described above, an optical density of 0.65 was considered to represent 100% CaSE. Then, the CaSE solution was diluted with serum-free MEM for further analysis. The pH of 2% CaSE and 5% CaSE was 7.3 ± 0.06 and 7.7 ± 0.08, respectively.

### Enzyme-Linked Immunosorbent Assay

Δ^9^-THC concentration in CaSE was analyzed by a direct competitive THC Forensic ELISA kit (NEOGEN^®^) according to manufacturer’s instructions. The concentration of interleukin-8 (IL-8) in the cell culture supernatant was determined by ELISA (Human IL-8 ELISA Duo Set, R&D Systems, United States) according to the manufacturer’s instructions. The absorbance was read at 450 and 570 nm within 15 minutes by infinite TECAN (M200 pro, TECAN, CA).

### Cell Culture and Transfection

Human embryonic kidney (HEK) 293 cells were cultured in DMEM supplemented with 10% fetal bovine serum (FBS) and gentamicin (20 μg/ml). Cells were grown at 37°C in 5% CO_2_ and 90% humidity. HEK293 cells were seeded at a density of 1 × 10^6^ cells per 100-mm dish and transfected the next day with 3 µg of sp-hCB1 with 120 ng of PKN-RBD-RlucII and 480 ng of rGFP-CAAX using PEI methods as described previously (Boussif et al., 1995; Namkung et al., 2018). Briefly, a total of 6 µg of DNA (adjusted with pcDNA3.1 zeo (+)) in 0.5 ml of PBS was mixed with 12 µl of PEI (25 kDa linear, 1 mg/ml) in 0.5 ml PBS and then incubated for 20 min at RT prior to applying to the cells. After 24 h, cells were detached and seeded onto poly-ornithine-coated 96-well white plates at a density of 25,000 cells per well for the BRET assays, which were performed 48 h after transfection.

Primary human lung fibroblasts (HLFs) were isolated from cancer-free lung tissue by explant procedure as described (Baglole et al., 2005). This study was approved by the Research Ethics Board of St. Joseph’s Healthcare Hamilton and informed written consent was obtained from each patient. Experiments were conducted with fibroblasts from three different individuals of the non-smoker group (Normal; M/F = 1/2; age 68 ± 9 years) and within passage six to nine. HLFs were cultured in 10% MEM and treated with THC dominant CaSE for 6 and 24 h.

### Rho BRET Assay

BRET assay for detecting Rho activation was performed as previously described (Namkung et al., 2018). Briefly, cells in 96 well plates were washed once with 150 µl/well of Tyrode’s buffer (140 mM NaCl, 2.7 mM KCl, 1 mM CaCl_2_, 12 mM NaHCO_3_, 5.6 mM D-glucose, 0.5 mM MgCl_2_, 0.37 mM NaH_2_PO_4_, 25 mM HEPES, pH 7.4) and left in 80 µl/well of Tyrode’s buffer. 2-AG, THC, and CBD were serially diluted in 15% MeOH in Tyrode’s buffer. The final concentration of MeOH in the assay is 3.75%. For BRET assay, the cells were loaded with 10 µl of coelenterazine 400a (final concentrations of ∼3.5 µM) and then the cells were stimulated with 30 µl of ligands or two-fold diluted CaSE in Tyrode’s buffer for 4 min prior to BRET measurement. Thus, final concentrations of CaSE were 12.5% (8-fold dilution of original CaSE). BRET signals were measured using a Synergy2 (BioTek) microplate reader. The filter was set at 410/80 nm and 515/30 nm for detecting the RlucII *Renilla* luciferase (donor) and rGFP (acceptor) light emissions, respectively. The BRET ratio was determined by calculating the ratio of the light emitted by rGFP over the light emitted by the RlucII.

### Liquid Chromatography With Tandem Mass Spectrometry

CaSE culture media samples were diluted 1:20 v/v by adding 10 µl to 190 µl of MeOH containing an internal standard CBD-d9 (10 pmol); 10 µl was subsequently analyzed by LC-MS/MS. In some cases, a 1:2 dilution was prepared by mixing 100 µl of CaSE culture medium with 100 µl of methanol containing internal standard CBD-d9 (10 pmol). CBD was chromatographed on a Waters UPLC reversed phase column (100 × 2.1 mm i.d.) using a blend of water and acetonitrile containing 0.1% acetic acid with a flow rate of 0.2 ml/min. The eluate was directed into a Thermo Quantum Access Max triple-quadrupole mass spectrometer and the CBD and CBD-d9 detected by single-reaction monitoring. The peak area for CBD was normalized by the peak area for the internal standard (CBD-d9) and the ratio compared to an external calibration curve for CBD prepared in MeOH. The limit of quantitation for CBD was 10 nM.

### Western Blot

HLFs were grown to approximately 70–80% confluence and cultured with serum-free MEM for 18 h before the treatment. Total cellular protein was extracted using RIPA lysis buffer (Thermo Scientific, Rockford) containing Protease Inhibitor Cocktail (PIC, Roche, United States). Ten to 20 μg of protein lysate were subjected to 10% SDS-PAGE gels and transferred onto Immuno-blot PVDF membranes (Bio-Rad Laboratories, Hercules, CA). Then, the membrane was blocked for 1 hour at room temperature in blocking solution (5% w/v of non-fat dry milk in 1x PBS/0.1% Tween-20). The primary antibodies, COX-2 (1:1,000; Cell Signaling Technology, CA) and β-Tubulin (1:50000; Sigma, CA) were added to the membranes and incubated overnight at 4°C or 1 h at room temperature. After several washes, membrane was incubated with secondary antibodies goat anti-rabbit IgG HRP-linked (1:10000, Cell Signaling Technology, CA) or HRP-conjugated horse anti-mouse IgG (1:10000, Cell Signaling Technology, CA). Detection of protein was done by enhanced chemiluminescence (ECL) and visualized using a ChemiDoc™ MP Imaging System (Bio-Rad, CA). Densitometric analysis was performed using Image Lab™ Software Version 5 (Bio-Rad, CA). Protein expression was normalized to β-tubulin and the data presented as the fold-change relative to the untreated condition.

### Quantitative RT-PCR

Using the Aurum™ Total RNA Kit (Bio-Rad, CA), total RNA was isolated according to the manufacturer’s instructions. Quantification of RNA was conducted on a Nanodrop 1,000 spectrophotometer. Reverse transcription of RNA was carried out using iScript™ Reverse Transcription Supermix (Bio-Rad, CA). Then, using this cDNA template, mRNA levels of *PTGS2*, *CXCL8* and *S9* were analyzed by quantitative PCR (qPCR) by using 1 µl of cDNA (10 ng/μl) and 0.5 µM primers with SsoFast™ EvaGreen^®^ (Bio-Rad, CA). Sequences of gene-specific primers are listed in Table 1. PCR amplification was performed using a CFX96 Real-Time PCR Detection System (Bio-Rad, CA). Thermal cycling was initiated at 95°C for 3 min and followed by 39 cycles denaturation at 95°C for 10 s and annealing at 59°C for 5 s. Gene expression was analyzed using the ΔΔC_T_ method, and results are presented as fold-change normalized to housekeeping gene (*S9*).

**TABLE 1:** Primer sequences used for qRT-PCR analysis.

Gene	Forward Primer Sequence	Reverse Primer Sequence
*PTGS2*	TCA CAG GCT TCC ATT GAC CAG	CCG AGG CTT TTC TAC CAG A
*CXCL8*	GAT GTC AGT GCA TAA AGA CAT ACT CCA A	GCT CTC TTC CAT CAG AAA GCT TTA CAA TA
*S9*	CAG CTT CAT CTT GCC CTC A	CTG CTG ACG CTT GAT GAG AA

### Statistical Analysis

Using GraphPad Prism 6 (v. 6.02; La Jolla, CA), statistical analysis was performed using a one-way analysis of variance (ANOVA) followed by Dunn’s multiple comparisons test to assess differences between treatments. Groups of two were analyzed by paired t-test. A two-way analysis of variance (ANOVA), followed by Tukey’s multiple comparisons test was used to evaluate differences between groups and conditions of more than two. Results are presented as mean ± standard error of the mean (SEM) or as mean ± standard deviation (SD) of the fold-changes compared to control cells. Experimental readings were done in triplicate and averaged; statistical analysis was therefore done using averaged values from three to five independent experiments unless otherwise indicated. In all cases, a *p* value <0.05 is considered statistically significant. For Tables 4, 5, the standard THC concentration response curve was obtained from a nonlinear regression curve fitting in GraphPad Prism software. The mean, upper limits, and lower limits of the unknowns were interpolated from the fitted standard curve with a confidential interval of 95%.

## Results

### Generation of Cannabis Smoke Extract Preparations That Contains Δ^9^-THC and CBD

**FIGURE 1:**
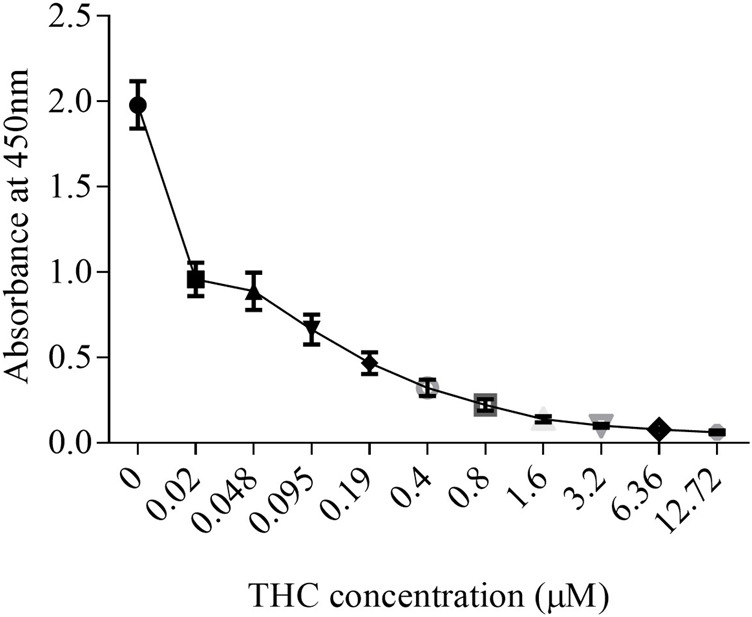
The standard curve for Δ^9^-THC. Δ^9^-THC was diluted in ELISA buffer from a starting concentration of 1 mg/ml to an upper limit of 4 μg/ml. Then, the concentration of this Δ^9^-THC standard curve was ranged from 0 μg/ml (buffer only)- 4 μg/ml (0–12.7 µM). Results are expressed as the mean ± SEM of two to four independent experiments.

Like tobacco, cannabis smoke contains hundreds of combustion products. However, cannabis also contains cannabinoids that exert biological and pharmacological effects. Standardized preparations of aqueous cigarette smoke extract (CSE) are well-described in the literature and are used to understand the consequences of tobacco exposure (Carnevali et al., 2003; Baglole et al., 2006; Baglole et al., 2008b; Hecht et al., 2014); no such standardized extract for cannabis smoke exists. Moreover, CSE prepared in cell culture media or PBS contains water soluble gas and particle phases of cigarette smoke (Kim et al., 2018). While many of these same compounds would be captured from cannabis smoke, cannabis also contains cannabinoids which are hydrophobic (Huestis, 2007) and unlikely to be present in an aqueous extract suitable for *in vitro* testing. Therefore, we sought to develop a cannabis smoke extract (CaSE) that contains biologically active cannabinoids. First, we utilized a semi-quantitative THC Forensic ELISA kit for which we developed a standard curve using Δ^9^-THC to allow for subsequent quantification. The standard curve was first prepared to calculate the relative concentration of Δ^9^-THC relative to the absorbance. We diluted Δ^9^-THC (in the ELISA buffer) from a starting concentration of 1 mg/ml to an upper limit of 4 μg/ml. The concentration of this Δ^9^-THC standard curve therefore ranged from 0 μg/ml (buffer only)- 4 μg/ml (0–12.7 µM) (Figure 1) and was used for analysis with all CaSE preparations.

Next, we evaluated Δ^9^-THC level by ELISA and CBD level LC-MS/MS in various aqueous CaSE preparations. Given that cannabinoids are hydrophobic, we compared Δ^9^-THC levels in CaSE prepared in standard cell culture media with and without MeOH. As additional controls, we also evaluated Δ^9^-THC concentrations in CSE prepared from research grade cigarettes. As expected, cell culture media alone with 30% MeOH as well as CSE (with 30% MeOH) contained no Δ^9^-THC or CBD (Table 2). We also measured Δ^9^-THC and CBD concentrations in CaSE prepared from the different strains of cannabis with reported varying amounts of Δ^9^-THC and CBD. Δ^9^-THC levels in CaSE generated from the Δ^9^-THC dominant and THC/CBD balanced strains with 10% MeOH were 0.62 ± 0.2 and 0.36 ± 0.02, respectively, and were therefore similar to the level in CaSE without MeOH. However, in CaSE generated from the Δ^9^-THC dominant strain with 30% MeOH, there were significantly higher levels of Δ^9^-THC compared to the CaSE without MeOH (THC dominant CaSE; Table 2). CBD levels were below the limit of detection by LC-MS/MS. Here, the estimated Δ^9^-THC concentration was 6.7 ± 0.29 µM in CaSE prepared in cell culture media with 30% MeOH. Preparation of CaSE from the balanced cannabis strain with 5–10% THC and 5–11% CBD also yielded significant Δ^9^-THC levels only when CaSE was prepared in media containing 30% MeOH. Finally, CaSE prepared from the CBD dominant cannabis strain in media with 30% MeOH has less Δ^9^-THC compared to CaSE prepared from the other two cannabis strains (Table 2). In CaSE generated from the balanced cannabis strain with 5–10% THC and 5–11% CBD, there were higher levels of CBD compared to the CaSE without MeOH (THC/CBD balanced CaSE; Table 2). CaSE prepared from the CBD dominant cannabis strain in media with 30% MeOH has higher CBD compared to CaSE prepared from the THC dominant strains. However, CBD levels are similar between CBD dominant and THC/CBD balanced strains (Table 2). We also generated CaSE from the Δ^9^-THC dominant strain in media with 30% EtOH. We found that the Δ^9^-THC level was slightly less in CaSE containing EtOH (5.7 ± 0.35 µM) comparing to CaSE with MeOH (6.7 ± 0.29 µM). These data show that preparation of CaSE in cell culture media with MeOH yields significantly higher concentrations of Δ^9^-THC and CBD compared to CaSE prepared without MeOH. Thus, the remainder of experiments were conducted with CaSE prepared in media with 30% MeOH and is refereed to hereafter as CaSE.

**TABLE 2:** Estimated concentration of Δ^9^-THC and CBD in CaSE.

Extract	THC Absorbance (ELISA)	Δ^9^-THC (µM) (ELISA)	CBD (µM) (LC-MS/MS)
Media +30% MeOH	1.662	0	<0.01
CSE+ 30% MeOH	1.398	0	<0.01
THC dominant CaSE	0.29 ± 0.02	0.67 ± 0.05	<0.01
THC dominant CaSE +30% MeOH	0.08 ± 0.002	6.7 ± 0.29*	<0.01
THC/CBD balanced CaSE	0.4538 ± 0.037	0.34 ± 0.05	<0.01
THC/CBD balanced CaSE +30% MeOH	0.087 ± 0.008	5.5 ± 0.46**	10.31 ± 9.125
CBD dominant CaSE+ 30% MeOH	0.16 ± 0.01	1.7 ± 0.4	7.733 ± 2.652

*THC dominant CaSE+30% MeOH was significantly higher (*p* < 0.03) compared to THC dominant CaSE without MeOH.; **THC/CBD balanced CaSE+30% MeOH was significantly higher (*p* < 0.008) compared to THC/CBD balanced CaSE without MeOH., Results are expressed as the mean ± SEM, of three to five independent extracts.

### Standardization of Cannabis Smoke Extract Using OD_320_

The tar components in tobacco and cannabis are similar (Tashkin, 2013), and chemical species of tobacco tar absorb light at 320 nm (Taylor et al., 2020). Thus, to ensure consistency in CaSE preparations between experiments, we standardized each CaSE preparation as previously described for CSE (Baglole et al., 2008a; Zago et al., 2013; Guerrina et al., 2021a; Guerrina et al., 2021b). Nine extracts from THC dominant cannabis were prepared and two measurements were taken for fresh extracts and after thawing of the same extracts that had been frozen at −80°C for 16 weeks. The optical density (OD) at 320 was 0.7 ± 0.05 and 0.64 ± 0.05 for fresh and frozen extracts, respectively (Table 3). Given that an OD of 0.65 is used to represent 100% CaSE, the percentage of CaSE averaged to be 110% ± 8 and 99% ± 7.7 for fresh and frozen extracts, respectively. We also evaluated Δ^9^-THC content by ELISA. The estimated Δ^9^-THC concentration of fresh and frozen extracts was similar and was approximately 12 µM. These data suggest that storage of CaSE extracts up to 16 weeks at −80°C does not affect Δ^9^-THC concentration and that an OD_320_ can be used to standardize aqueous CaSE to minimize batch-to-batch variability.

**TABLE 3:** Δ^9^-THC absorbance (OD_320_) and estimated concentration by ELISA.

Extract	OD_320_	Percentage	THC ELISA	Δ^9^-THC (µM)
Fresh CaSE	0.7 ± 0.05	110% ± 8	0.06 ± 0.0005	12.4 ± 0.2
Frozen CaSE	0.64 ± 0.05	99% ± 7.7	0.066 ± 0.001	12.2 ± 0.2

Results presented as mean ± SEM, of 9 independent extracts.

### Cannabis Smoke Extract Activates CB1 Receptors

**FIGURE 2:**
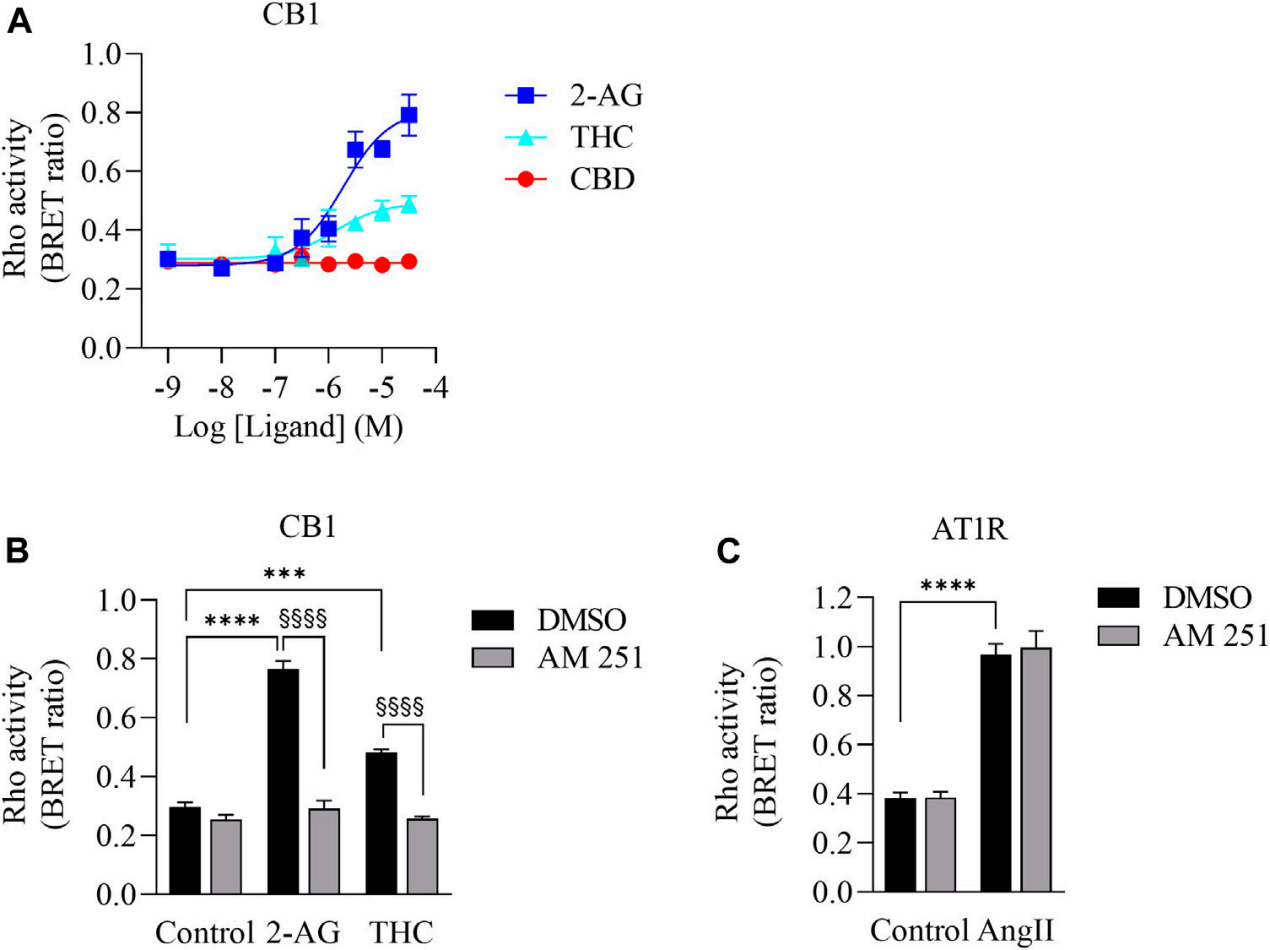
Validation of CB1-mediated Rho activation. **(A)**. Concentration response curves of Rho activation in HEK293 cells expressing CB1, PKN-RBD-RLucII and rGFP-CAAX. Cells were stimulated with either 2-AG (blue square), THC (turquoise triangle) or CBD (red circle). CB1 was activated with 2-AG and Δ^9^-THC but not with CBD. Data represent means ± SEM of four independent experiments performed in triplicate. **(B)**. Validation of CB1-mediated Rho activation by CB1 antagonist AM-251. Cells were stimulated with control, 2-AG (10 µM) or Δ^9^-THC (THC, 10 µM) in the absence (vehicle, 0.1% DMSO (black bar)) or presence of 10 µM of AM-251 (grey bar). There was an increase in Rho activation in cells exposed to 2-AG (****p < 0.0001) and Δ^9^-THC (***p < 0.0002). AM251 abolished 2-AG- and THC-induced CB1 activation (^§§§§^ *p* < 0.0001). **(C)**. Cells expressing AT1R, PKN-RBD-RLucII and rGFP-CAAX were stimulated with control or with 100 nM of AngII, agonist for AT1R, with 0.1% of DMSO (black bar) or 10 µM of AM-251 (grey bar). There was an increase in AT1R-mediated Rho activation in cells exposed to AngII (****p < 0.0001). There was no effect of AM251 on AT1R-mediated Rho activation. Data represent means ± SEM of three independent experiments.

Δ^9^-THC has high affinity to CB1 and CB2 receptors (Pertwee, 2010), which are G protein coupled receptors (GPCRs). CB1 couples to not only G_i/o_ but also to the G_12/13_ subfamily and activates the down-stream protein Rho (Inoue et al., 2019; Krishna Kumar et al., 2019; Avet et al., 2020). To determine whether there is sufficient Δ^9^-THC in the CaSE preparations to activate CB1, we used a BRET-based Rho biosensor (Namkung et al., 2018). We transiently transfected HEK293 cells with signal-peptide-human CB1 (CB1) along with PKN-RBD-RLucII and rGFP-CAAX (Rho sensor) and stimulated the cells with Δ^9^-THC, CBD and 2-arachidonoylglycerol (2-AG), an endogenous CB ligand (Figure 2). The BRET signal increased in response to Δ^9^-THC and 2-AG but not to CBD (Figure 2A). Further, we observed that AM251, a CB1-specific antagonist, abolished the THC- and 2-AG- promoted BRET signals (Figure 2B). To verify the specificity of AM251 on CB1-mediated Rho activation, we examined the effect of AM251 on angiotensin II type 1 receptor (AT1R)-mediated Rho activation, which also couple to this pathway (Namkung et al., 2018). AM251 showed no effect on the basal BRET whereas AngII induced a BRET signal in HEK293 cells expressing AT1R along with Rho sensor (Figure 2C). These data show that Δ^9^-THC- and 2-AG- promoted CB1 activation and signaling to the G_12/13_-Rho pathway.

**FIGURE 3:**
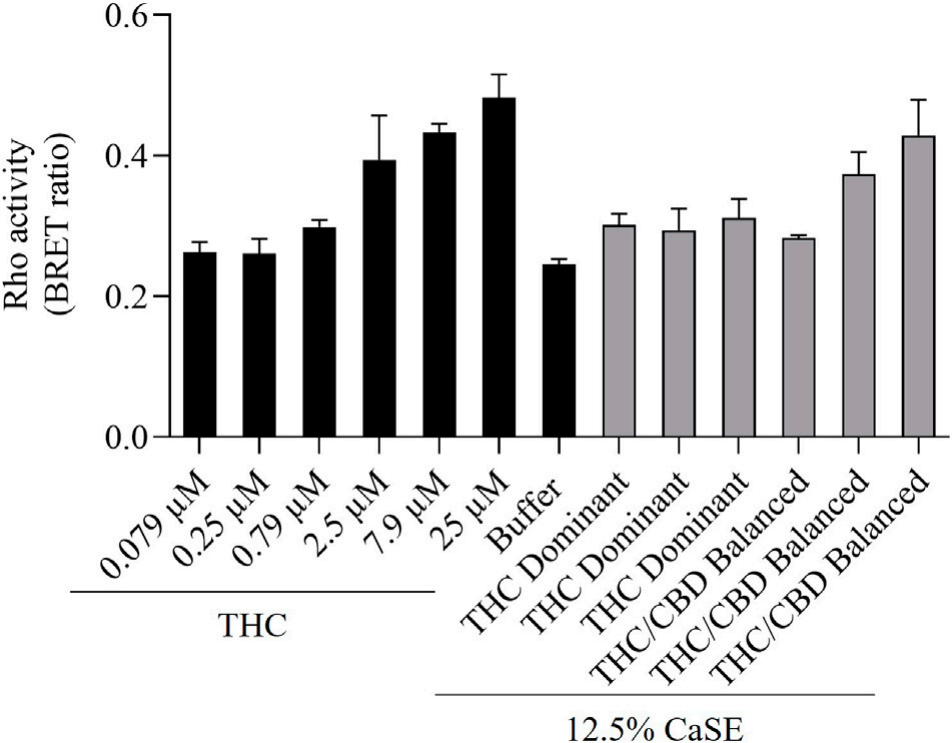
CaSE promotes Rho activation in CB1 expressing cells. HEK293 cells expressing CB1 along with PKN-RBD-RLucII and rGFP-CAAX were stimulated with indicated concentrations of Δ^9^-THC in buffer or 8-fold diluted CaSE (15 µl in total 120 µl assay volume, 12.5% CaSE) from Δ^9^-THC dominant and THC/CBD balanced strains prepared in media with 30% MeOH. There was an increase in CB1 activation in a concentration-dependent manner by Δ^9^-THC. There was an increase in the activation of CB1 in cells treated with CaSE from THC dominant or THC/CBD balanced cannabis. Buffer was 8-fold dilution of 30% MeOH/DMEM with Tyrode’s buffer. Data represent means ± SD of triplicate (THC) and duplicate (CaSE) of a representative experiment. Similar results were obtained with 20 µl or 10 µl application of CaSE.

We next vetted three different extracts prepared from THC dominant or THC/CBD balanced cannabis prepared in media with or without 30% MeOH to verify that these CaSE preparations contained biologically active Δ^9^-THC; we utilized the same extracts as for the data presented in Table 2. First, the activation of CB1 in response to different concentrations of Δ^9^-THC (0.3–25 µM) was assessed. There was a concentration-dependent activation of CB1 by Δ^9^-THC (Figure 3). Furthermore, there was an increase in CB1 activation in cells treated with CaSE from THC dominant or THC/CBD balanced cannabis prepared in media with 30% MeOH (Figure 3). Extracts in media without 30% MeOH did not show BRET signals in our assay (data not shown). Based on CB1 activation by Δ^9^-THC (Figure 3), we extrapolated that CaSE prepared from THC-dominant cannabis activates CB1 in concentrations equivalent to 5–7 µM of Δ^9^-THC (Table 4). CaSE from THC/CBD balanced cannabis also activates the receptor, which is equivalent to 3–50 µM of Δ^9^-THC (Table 4).

**TABLE 4:** Estimation of THC concentrations in 100% CaSE. THC concentrations in CaSE were estimated from interpolation of standard THC concentration response curve in Figure 3.

CaSE	THC conc. (μM)	95% CI
THC dominant	5.32	3.47–8.31
THC dominant	4.45	2.77–7.04
THC dominant	6.58	4.43–10.14
THC/CBD balanced	3.23	1.67–5.30
THC/CBD balanced	18.83	12.80–27.10
THC/CBD balanced	47.16	31.88–73.00

**FIGURE 4:**
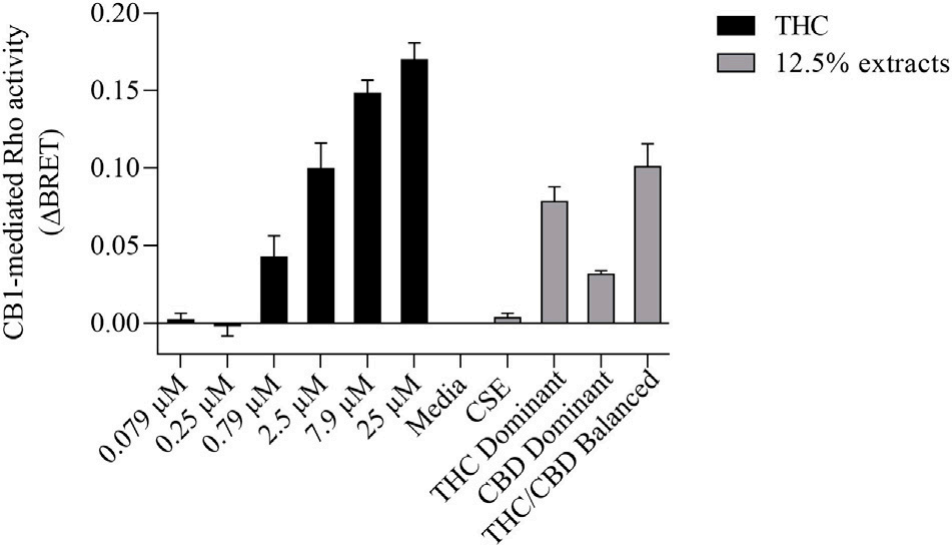
CaSE promotes Rho activation in CB1 expressing cells in comparison to Δ^9^-THC. HEK293 cells expressing CB1 along with Rho sensor were stimulated with indicated concentrations of Δ^9^-THC in buffer or 8-fold diluted indicated extracts prepared in media with 30% MeOH: Media with only 30% MeOH, CaSE and CSE. There was an increase in CB1 activation in cells exposed to CaSE from THC dominant, THC/CBD balanced and CBD-dominant cannabis, but not Media with MeOH or CSE. Buffer was 8-fold dilution of 30% MeOH/DMEM with Tyrode’s buffer. Data were expressed as a ligand-promoted BRET (ΔBRET) by subtracting BRET ratio in control media. Data represent mean ± SEM of three to five independent experiments.

**FIGURE 5:**
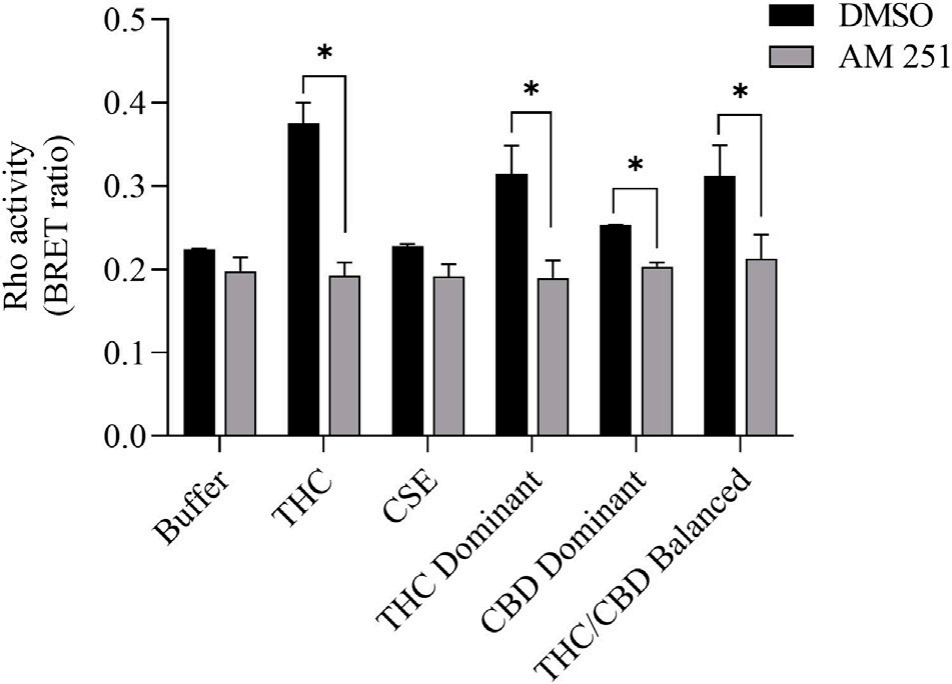
CaSE-induced Rho activation is mediated by CB1. HEK293 cells expressing CB1 and Rho sensor were stimulated with Δ^9^-THC (25 µM) or indicated CaSE (12.5%) in the absence (0.1% DMSO, black bar) or presence of AM 251 (10 µM) (grey bar). AM 251 abolished the Δ^9^-THC- and CaSE-mediated Rho activation in CaSE prepared from THC dominant and THC/CBD balanced strains (*p < 0.05). CSE treatment did not increase Rho activity compared to buffer; AM 251 had no effect. Data represent mean ± SD from two independent experiments.

We then tested whether the receptor itself was affected by the MeOH and evaluated the specificity of the system by adding CSE prepared in media with 30% MeOH; we also included CaSE from all three cannabis strains (see Table 2). We found that there was no Rho activation with media containing 30% MeOH or CSE (Figure 4). CaSE from THC dominant, THC/CBD balanced, and CBD-dominant cannabis all activated Rho signaling (Figure 4), at levels that corresponded approximately to between 4–22 µM of Δ^9^-THC present in the extracts (Table 5). Thus, CaSE, but not media containing MeOH or CSE, activates the CB1 receptor. Finally, we used the CB1 antagonist AM251 to confirm that CaSE is specific in its ability to activate CB1. AM251 inhibited THC-induced Rho activation. We also found that AM251 significantly inhibits CaSE-induced Rho activation for the CaSE prepared from the THC-dominant and THC/CBD balanced strains (Figure 5). Thus, CaSE induces Rho activation through CB1. Taken together, these data show that a standardized preparation of CaSE contains biologically active cannabinoids.

**TABLE 5:** Estimation of THC concentration in 100% CaSE. THC concentrations in CaSE were estimated from interpolation of standard THC concentration response curve in Figure 4.

CaSE	Est. Concentration (μM)	95% CI
THC dominant	13.9	10.0–19.5
THC/CBD balanced	22.1	15.7–30.3
CBD dominant	4.5	3.0–6.7

### COX-2 and IL-8 Are Increased in HLFs Exposed to Cannabis Smoke Extract

**FIGURE 6:**
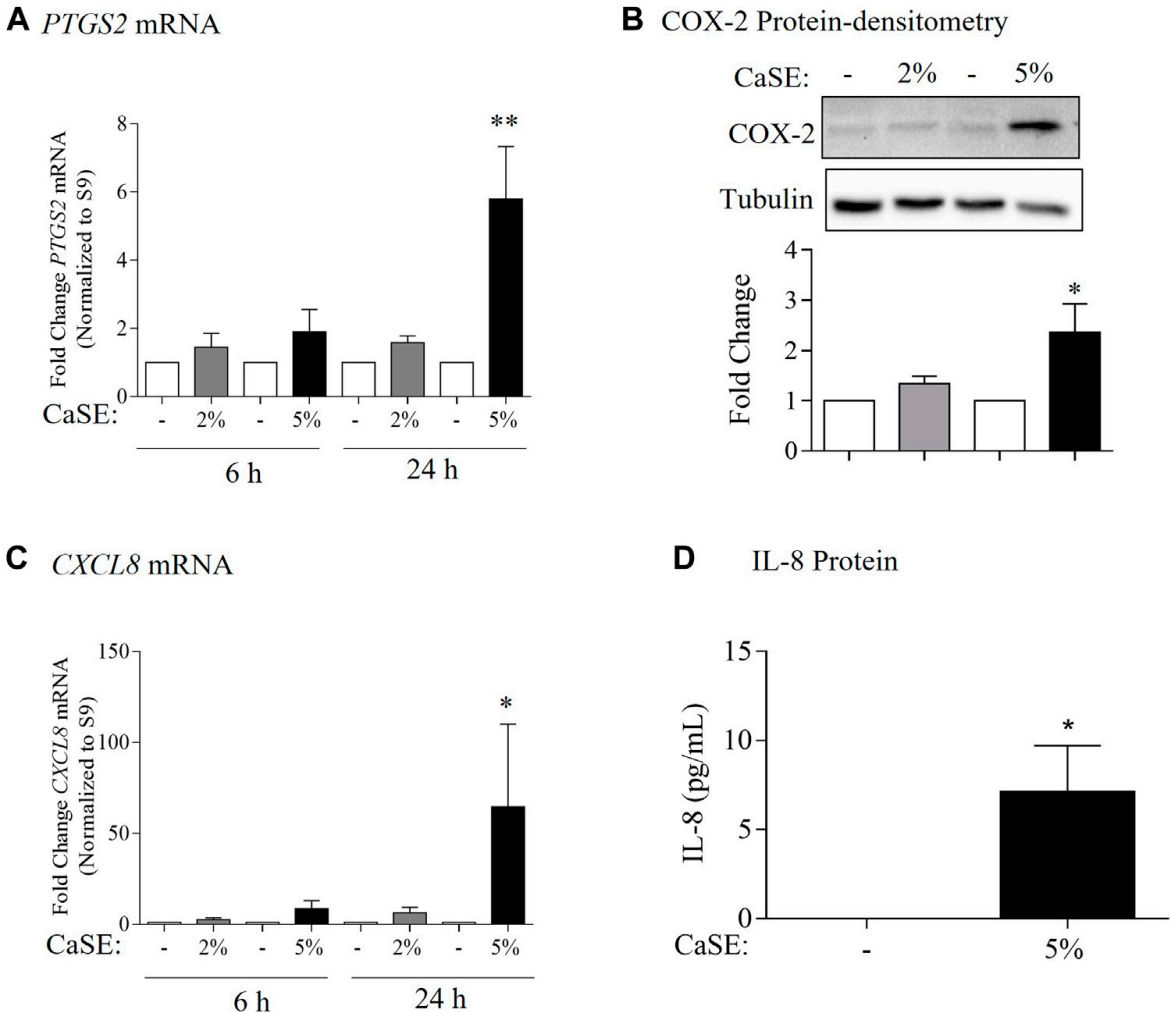
CaSE induces COX-2 and IL-8 expression in human lung fibroblasts. **(A)**. *PTGS2* mRNA: there was a slight increase, but not statistically significant, in *PTGS2* mRNA in HLFs exposed to 2 and 5% CaSE for 6 h and in HLFs exposed to 2% CaSE for 24 h compared to corresponding control. There was significant increase in *PTGS2* mRNA in HLFs exposed to 5% CaSE for 24 h (**p = 0.009) compared to corresponding control. Results are expressed as the mean ± SEM of 4 independent experiments of HLFs used from 3 Normal subjects. **(B)**. COX-2 Protein-densitometry: there was significant increase in COX-2 protein levels in HLFs exposed to 5% CaSE for 24 h (*p = 0.04) compared to corresponding control. Results are expressed as the mean ± SEM of 3 independent experiments (HLFs used from 3 Normal subjects). **(C)**. *CXCL8* mRNA: there was a slight -but not statistically significant-increase in *CXCL8* mRNA in HLFs exposed to 2 and 5% CaSE for 6 h There was significant increase in *CXCL8* mRNA in HLFs exposed to 5% CaSE for 24 h (*p = 0.01) compared to corresponding control. Results are expressed as the mean ± SEM of 4 independent experiments (HLFs used from 3 Normal subjects). **(D)**. IL-8 Protein: there was an increase in IL-8 protein levels in the media from HLFs exposed to 5% CaSE for 24 h compared to corresponding control. Results are expressed as the mean ± SEM of 3 independent experiments (HLFs used from 3 Normal subjects).

COX-2 and IL-8 are among the proinflammatory mediators that are induced by tobacco smoke (Martey et al., 2004; Li et al., 2007). IL-8 is also elevated in serum from cannabis smokers (Bayazit et al., 2017). To explore whether we could replicate these findings, we characterized the effect of CaSE exposure on the expression of COX-2 and IL-8 at the mRNA and protein levels in primary HLFs. For these experiments, HLFs were treated with either 2% or 5% CaSE that was prepared from THC dominant cannabis. Selection of these concentrations was based on our previous publications with CSE (Baglole et al., 2008a; Guerrina et al., 2021a). These concentrations of CaSE did not affect cell viability (data not shown). The concentration of Δ^9^-THC in 2% CaSE was 0.18 ± 0.003 µM and in 5% CaSE was 0.45 ± 0.006 µM (n = 3). The mRNA for *PTGS2* did not increase with 6 h of CaSE (Figure 6A). However, there was a significant increase in *PTGS2* mRNA upon exposure to 5% CaSE for 24 h- but not 2% CaSE. Accordingly, there was a significant increase in COX-2 protein with 5% CaSE (Figure 6B). There was also a significant increase in *CXCL8* mRNA in response to 5% CaSE for 24 h (Figure 6C). At the protein level, IL-8 was also induced upon 5% CaSE treatment for 24 h (Figure 6D). These data indicate that a standardized CaSE preparation, containing biologically active cannabinoids, induces an inflammatory response in primary HLFs.

## Discussion

Cannabis is the most commonly-smoked plant after tobacco (Baron, 2018; Brown, 2020; Campeny et al., 2020; Li et al., 2020). Recently, the personal use of cannabis has been approved in nine states of the United States as well as in Uruguay and Canada (Campeny et al., 2020). Cannabis smoke is often considered to be harmless compared to tobacco smoke (Sinclair et al., 2013). However, cannabis smoke contains many chemicals (toxicants, irritants, carcinogens, and fine particles) as does tobacco smoke (Moir et al., 2008; Manolis et al., 2019; Graves et al., 2020). The latest report from the Canadian Centre on Substance Use and Addiction (CCSA) highlights the risks of cannabis smoking to the heart and lungs as heavy users of cannabis can potentially develop cardiovascular and respiratory diseases (Canadian Centre on Substance Use and Addiction, 2020). Cannabis smoking is associated with a greater incidence of respiratory symptoms including sore throat, productive cough and shortness of breath (Henderson et al., 1972). These symptoms are likely due to harmful impacts of cannabis smoke on the respiratory system. Indeed, there is evidence of goblet cell hyperplasia, squamous metaplasia and inflammation in tracheobronchial specimens of cannabis smokers compared to non-smokers (Fligiel et al., 1997) as well as airway inflammatory changes in asymptomatic marijuana smokers compared to non-smokers (Roth et al., 1998). This is also supported by *in vivo* studies which showed that exposing mice to cannabis smoke alters the immune cell populations in the airways and lung tissue (Fantauzzi et al., 2021) and induces bronchial hyperreactivity, inflammation, and tissue destruction (Helyes et al., 2017). Thus, cannabis smoke may cause adverse respiratory features, and may increase the risk of developing lung diseases similar to tobacco smoke. However, the number of studies investigating the health effects of cannabis smoke exposure remains limited, and it is not well understood if there is a link between exposure to cannabis smoke and respiratory disease development. Thus, there is a need for experimental models into order to investigate the impact of cannabis smoke on respiratory health.

Despite this need, there are no validated experimental models with which to perform detailed evaluations on the effect of cannabis smoke *in vitro*. We are only aware of one study utilizing a cannabis smoke extract for *in vitro* assessment (Aguiar et al., 2019). However, the cannabis smoke extract in that study was prepared without adding a solvent to capture the cannabinoids in the aqueous solution; the presence of Δ^9^-THC or other cannabinoids was also not measured (Aguiar et al., 2019). Based on our results, an aqueous preparation of cannabis smoke-as in the study by Aguiar and colleagues-likely did not contain active cannabinoids. Therefore, we sought to develop a standardized protocol for the preparation of CaSE utilizing a protocol similar to that used in the generation of CSE (Baglole et al., 2008a; Zago et al., 2013; Guerrina et al., 2021a; Guerrina et al., 2021b) but one that contains cannabinoids. To achieve this, we made a modification to the preparation via the addition of MeOH to the cell culture media, as cannabinoids are hydrophobic (Huestis, 2007) and MeOH is a suitable solvent for the isolation of fat-soluble compounds (Rozanc et al., 2021). Thus, the addition of MeOH significantly increased the concentration of Δ^9^-THC and CBD in the extract compared to negligible levels in CaSE prepared in culture media alone. One of the advantages of this standardized method is that it can be performed using common laboratory equipment, allowing for easy adaptation. Here, we followed the same standardization method for CSE by measuring the absorbance of CaSE at 320 nm, similar to what we have previously used for CSE (Martey et al., 2004; Baglole et al., 2008a; Zago et al., 2013; Zago et al., 2014). Because the tar components in tobacco and cannabis are similar (Tashkin, 2013) and the chemical species of tar in tobacco absorb light at 320 nm (Taylor et al., 2020), standardization can be performed via spectroscopy, and confirmation of cannabinoid presence made by a commercial ELISA. One of the limitations of this study is that we measured only Δ^9^-THC and CBD levels in CaSE, and thus cannot provide information on the presence or absence of additional cannabinoids or other compounds, including those could also affect the activity of the CB1 receptor. Another limitation that we did not assess whether MeOH affects the solubility of the chemical species found in the tar fraction. Nonetheless, this methodology allows for robust and reliable generation of a cannabis extract that contains biologically-active cannabinoids (Δ^9^-THC and CBD) to allow for consistency between experiments and comparison between studies.

The detection of cannabinoids in CaSE is important as cannabinoids carry out a variety of physiological functions by engaging with receptors present in the body, including cannabinoids receptors (CBR) (Reggio, 2010). The first discovered CBRs are CB1 and CB2, which belong to the GPCR superfamily. CB1 is expressed predominantly in the central nervous system (CNS), particularly in the basal ganglia, hippocampus, cortex, and cerebellum; these CB1 receptors mediate the psychoactive effects from Δ^9^-THC (Sim-Selley, 2003; Kawamura et al., 2006). Δ^9^-THC also binds to CB2 receptors with similar binding affinity (Pertwee, 2010). CB2 receptors are present mainly on the surface of immune and hematopoietic cells (Graham et al., 2010). In the respiratory system, CB1 and CB2 receptors are both expressed on epithelial cells with alveolar type II cells displaying CB1 receptor and lung fibroblasts having CB2 receptor (Kicman et al., 2021). Although lung fibroblasts provide structure and support to the lungs by synthesizing and maintaining an extracellular matrix (ECM) (White, 2015), fibroblast activation also leads to the production of several cytokines and chemokines (Buckley et al., 2001; Davidson et al., 2021). The effects of CSE on lung fibroblasts is well-described by us and others (Carnevali et al., 2003; Martey et al., 2004; Baglole et al., 2006; Baglole et al., 2008a; Baglole et al., 2008b), making these a relevant lung cell type. Herein, we observed that the CaSE-like CSE-induces an inflammatory response in primary lung fibroblasts, including induction of COX-2 and IL-8 levels by 5% CaSE derived from the THC dominant strain. By our estimation, this preparation contains ∼0.45 µM of Δ^9^-THC, which is similar to the plasma levels of THC in cannabis smokers (∼ 1 µM) (Azorlosa et al., 1992). The ability of 5% CaSE to induce COX-2 and IL-8 expression occurred despite the presence of cannabinoids at physiologically-relevant concentrations. It could be that Δ^9^-THC itself induced the inflammatory response; this would be in line with another publication whereby COX-2 is induced by Δ^9^-THC in neurons and astroglial cells (Chen et al., 2013). It could also be that the cannabinoids present in the extract could not compensate for products of combustion-which promote an inflammatory response typified by the induction of COX-2 (Martey et al., 2004). Of note is the absence of CBD from extracts prepared from the THC dominant strain. CBD has anti-oxidative and anti-inflammatory properties (Atalay et al., 2019). Comparison of CaSE prepared from different cannabis strains (with varying THC/CBD ratios) may shed light on whether all CaSE preparations have the same inflammatory potential.

In order for Δ^9^-THC and CBD to be biologically active, the acidic precursors THCA and CBDA need to undergo decarboxylation, a process that is facilitated by combustion. Our standardized CaSE indeed contained forms of cannabinoids that activated the CB1 receptor. As the CB1 receptor is coupled to G_i/o_ and G_12/13_ subfamilies and activates its down-stream Rho (Inoue et al., 2019; Krishna Kumar et al., 2019; Avet et al., 2020), we transfected cells with CB1 receptors along with Rho sensor to evaluate CB1 receptor activation. Here, it was only with CaSE prepared with MeOH that activated the CB1 receptor, with highest activation in extracts from the THC/CBD balanced strain. This was surprising, given that CBD has relatively low affinity for the CB1 receptor (McPartland et al., 2015) and our result showed that pure CBD does not activate CB1. However, it is still possible that CBD may modulate the activity of the receptor (McPartland et al., 2015) or that CBD and/or other cannabinoids in the extract affects the binding of Δ^9^-THC to the CB1 receptor. We also found that the estimated Δ^9^-THC concentration in these extracts from the functional assay was 3–50 μM, which is higher than the estimated concentration from the ELISA (∼5.5 µM). Nonetheless, the presence of biologically-active cannabinoids in this CaSE preparation further highlights its utility in evaluating the physiological and pathological implications of cannabis smoke.

A limitation of this study is that we did not assess additional signaling mechanisms that may account for the induction of inflammation of CaSE or the ability of CaSE to activate other receptors. For example, Δ^9^-THC also binds to the CB2 receptor (Pertwee, 2010) with CB2 activation controlling inflammation and immune functions (Turcotte et al., 2016). Δ^9^-THC can also activate the nuclear factor-κB (NF-κB) pathway (Do et al., 2004), a transcription factor that regulates genes involved inflammation, such as COX-2 and IL-8 (Ahn and Aggarwal, 2005). As lung fibroblasts express the CB2 receptor (Kicman et al., 2021), it may be that CaSE induces inflammation via the activation of CB2 receptor and/or NF-κB. However, Δ^9^-THC can also activate other GPCRs such as GPR55 (Sharir and Abood, 2010) which is also expressed in the lung (Ryberg et al., 2007). Interestingly, agonist interaction with GPR55 can also activate NF-κB (Henstridge et al., 2010). However, direct regulation of cannabinoids on the activation of GPR55 still needs to be elucidated. Finally, one of the downstream signaling pathways of the CB1 receptor is p38 MAPK (Chen et al., 2013). It is well studied that cigarette smoke can also active p38 MAPK to induce an inflammatory response (Moretto et al., 2012; Marumo et al., 2014). However, nothing is known about the effect of cannabis smoke on this- and other-signaling pathways in pulmonary cells, a deficit in knowledge that can be addressed by utilization of this standardized extract.

In this study, we sought to develop a protocol for the preparation of a cannabis smoke extract that could be used to investigate the effect of cannabis smoke *in vitro*. We successfully captured Δ^9^-THC and CBD within an aqueous preparation (CaSE), which allowed us to recapitulate as closely as possible to what smokers are inhaling; this includes cannabinoids and combustion products. Our data also revealed that this CaSE activates CB1 receptors, further highlighting that it contains biologically active cannabinoids. Importantly, this extract can be prepared and standardized using common laboratory equipment. This CaSE can be used for further molecular investigation into the downstream mechanisms of cannabis smoke/cannabinoids that will ultimately improve our understanding about the effect of cannabis smoke on features of lung pathology.

## Data Availability

The authors acknowledge that the data presented in this study must be deposited and made publicly available in an acceptable repository, prior to publication.
